# Preparation of Pt Ag alloy nanoisland/graphene hybrid composites and its high stability and catalytic activity in methanol electro-oxidation

**DOI:** 10.1186/1556-276X-6-551

**Published:** 2011-10-07

**Authors:** Lili Feng, Guo Gao, Peng Huang, Xiansong Wang, Chunlei Zhang, Jiali Zhang, Shouwu Guo, Daxiang Cui

**Affiliations:** 1 Key Laboratory for Thin Film and Microfabrication Technology of Ministry of Education, National Key Laboratory of Micro/Nano Fabrication Technology, Research Institute of Micro/Nano Science and Technology, Shanghai Jiao Tong University, Shanghai 200240, P. R. China

## Abstract

In this article, PtAg alloy nanoislands/graphene hybrid composites were prepared based on the self-organization of Au@PtAg nanorods on graphene sheets. Graphite oxides (GO) were prepared and separated to individual sheets using Hummer's method. Graphene nano-sheets were prepared by chemical reduction with hydrazine. The prepared PtAg alloy nanomaterial and the hybrid composites with graphene were characterized by SEM, TEM, and zeta potential measurements. It is confirmed that the prepared Au@PtAg alloy nanorods/graphene hybrid composites own good catalytic function for methanol electro-oxidation by cyclic voltammograms measurements, and exhibited higher catalytic activity and more stability than pure Au@Pt nanorods and Au@AgPt alloy nanorods. In conclusion, the prepared PtAg alloy nanoislands/graphene hybrid composites own high stability and catalytic activity in methanol electro-oxidation, so that it is one kind of high-performance catalyst, and has great potential in applications such as methanol fuel cells in near future.

## Introduction

Graphene, a single-atom-thick sheet of hexagonally arrayed *sp*^2^-bonded carbon atoms, has attracted intensive interests in recent years [1], owing to its large specific surface area, high thermal and electrical conductivities [2-6], great mechanical strength [7]. The unique properties of graphene sheets provide applications in synthesis of nanocomposites [8-10], fabrication of field-effect transistors [11-13], dye-sensitized solar cells [14], lithium ion batteries [15,16], and electrochemical sensors [17]. Up to date, many methods such as a scotch tape (peel off) method [18], epitaxial growth [19,20], chemical vapor deposition [21], and reduction of graphene oxide [22-26] have been used to prepare individual graphene sheets and to improve the properties of graphene. Among these methods, chemical reduction method of graphene oxide is with lowest cost and large scale to prepare graphene, which attract scientists' intensive attention, and exhibit great application prospect.

In the field of electrochemistry, graphene is an excellent substrate to load active nanomaterials for energy applications due to its high conductivity, large surface area, flexibility, and chemical stability. For example, Dai and colleagues [15] made high-capacity anode material for lithium ion batteries by growing Mn_3_O_4 _nanoparticles (NPs) on graphene sheets. Zhang et al. [16] prepared mono-dispersed SnO_2 _NPs on both sides of single layer graphene sheets as anode materials in Li-ion batteries. They found much higher retention of SnO_2_-graphene composite than commercial SnO_2 _powder after 50 cycles. Apart from these studies, a lot of efforts had been paid on metal oxide/graphene hybrid composites [27]. However, so far, few reports are closely associated with the use of graphene-based metal materials as heterogeneous catalysts [28-30]. Therefore, to prepare and study graphene/noble metal, heterogeneous materials become more and more important.

In the field of catalysis, Pt (and Pd) is intensively applied in direct methanol fuel cells (DMFCs) [31,32], because of their high-efficient catalysis function for methanol dehydrogenation. To improve catalytic properties of the metal materials, the size and structure of NPs become more and more important. Pt NPs with several nanometers in diameter and porous structures own high catalytic activity because of their enlarged surface area. In addition, the composition of the catalyst is another important factor for catalytic activity. For instance, pure Pt nanostructures are easily poisoned by chemisorbed CO-like intermediates generated in the course of methanol oxidation, which makes their catalytic performance decreased quickly. To solve this problem, it is feasible to prepare bimetallic nanocomposites composed of Pt and those metals such as Ru, Rh, Pd, and Au [33-37]. Other metal materials are proposed to provide oxygen-containing species at relative negative potential, which can oxidize CO at Pt sites. Therefore, to prepare alloyed Pt NPs are very necessary. Wu and colleagues had proved that PtAg alloy nanoislands on gold nanorods had good optical responses and electrochemical catalytic activity [38,39]. However, up to date, graphene-based PtAg alloy nanoislands as heterogeneous catalysts are not still investigated well.

In this study, we reported to prepare PtAg alloy nanoislands/graphene hybrid composites based on the self-assembly of positively charged gold nanorods and Au@AgPt alloy nanorods on negatively charged graphene sheets. (Here "@" was defined as a core/shell structure. Au@AgPt alloy nanorod is a core/shell structure for Au nanorod as the core and AgPt alloy as the shell. We use Au@Pt_*m*_Ag_*n *_to represent the samples, and *m *and *n *are percentage determined by EDX.) The self-assembly technology enables loading a lot of Au NRs and Au@AgPt alloy nanorods on individual graphene sheets with uniform morphology. It was investigated that the prepared Au@AgPt alloy nanorods/graphene hybrid composites were used as a fuel cell electrocatalyst for methanol electro-oxidation. The utilization ratio of Pt was 23.4%, but its catalytic activity was 124 mA mg Pt^-1^, which was close to 162.5 mA mg Pt^-1 ^(99.2% utilization ratio of Pt) reported previously [40]. In addition, Pt material has also good catalytic stabilization, which shows that catalytic activity may increase with the utilization ratio of Pt increase, further investigation will be helpful to clarify its potential mechanism.

## Experimental section

### Chemicals

10000 mesh (dimension: 1.5µm) graphite, etyltrimethylammonium bromide (CTAB), PVP (K30, Mw = 30000-40000) were obtained from Alfa Company and used as-received. Sodium borohydride (NaBH_4_), chlorauric acid (HAuCl_4_·3H_2_O), silver nitrate (AgNO_3_), and potassium tetrachloroplatinate(II) (K_2_PtCl_4_), L-ascorbic acid (AA), methanol, sulfuric acid, potassium permanganate (KMnO_4_), hydrogenperoxide (H_2_O_2_), sodium nitrate (NaNO_3_), were purchased from Shanghai Sigma Company and used as-received. Milli-Q water (18 MΩ cm) was used for all solution preparations. All glassware used in the following procedures were cleaned in a bath of a piranha solution (H_2_SO_4_/30%H_2_O_2 _= 7:3 v/v) and boiling for 30 min.

### Synthesis

#### Synthesis of graphene nanosheets

Graphene oxides (GO) were synthesized from flake graphite (1.5 µm graphite) using modified Hummer's method [41,42]. Then graphite oxides were exfoliated by ultrasonication for more than 5 h. Well-dispersed homogeneous graphene oxide solution (0.5 mg mL^-1^) was obtained. PVP was used to prevent flocculation when reduced graphene oxide to graphene sheets. In a typical procedure for chemical conversion of graphene oxide to graphene (GN), 100 mL 8 mg mL^-1 ^PVP solution was added to 50 mL 0.5 mg mL^-1 ^GO solution, then stirred vigorously for more than 12 h. Afterward, 1.75 mL 0.5% hydrazine solution and 2 mL 2.5% ammonia solution were added. The mixture was stirred for 1 h at 95°C. After that, graphene was cooled at room temperature. The whole reduction process was repeated once more to reduce GO further. The stable black dispersion of GN was filtered under the condition of vacuum with 200 nm membrane as filter paper to collect it, at the same time it was washed with Milli-Q water (18 MΩ cm). Finally, the prepared GNs were dissolved in 50 mL water (0.5 mg mL^-1^).

#### Growth of Au@AgPt nanorods

Au@AgPt nanorods were prepared using an etching method described by Wu [38]. The specific process is consisted of four steps: (1) Au nanorods synthesis; (2) precoat a thin Pt layer on Au nanorod [43]; (3) grow Ag shell on Au@Pt NRs; and (4) etch Ag shell with Pt (II) ions.

#### Hybrid of graphene and Au nanorods

A certain volume of 0.5 mg mL^-1 ^GNs was added to 1 mL of the gold nanorods solution (0.5 mmol L^-1^) or Au@AgPt nanorods solution. The mixture solution was then shaken vigorously and sonicated for 30 s. Afterward, the mixture was left undisturbed and aged at room temperature for more than 24 h. The color of the solution changed from red (Au nanorods) or dark gray (Au@AgPt nanorods) to colorless, and the hybrid composites precipitated at the bottom of the vessel. Afterward, the precipitate was collected by centrifugation (12000 rpm for 5 min). Finally, the precipitate was redispersed in 100 µL water for electrochemical testing.

### Characterizations

UV-Vis-NIR absorption spectra were obtained from a Varian Cary 50 spectrophotometer. Scanning electron microscopy (SEM) images and energy dispersive X-ray (EDX) analysis were taken on a field emission scanning electron microscope (FESEM, Zeiss Ultra). Transmission electron microscopy (TEM) images were captured on a JEM-2010/INCA OXFORD at an accelerating voltage of 200 kV. Zeta potential results were carried out on zeta potential/particle sizer (Nicom 380ZLS). CHI660C electrochemical workstation (Chenhua, Shanghai) was carried out for the electrochemical measurement. Cyclic voltammetry was performed in a three-electrode glass cell at room temperature. Glassy carbon (GC) electrode was used as working electrode. Before testing, the electrode was rejuvenated by polished with 0.3 and 0.05 µm alumina powders, respectively, then sonicated sequentially in alcohol, pure water in each for about 20 min. 5 μL as-prepared samples were drop-casted onto GC electrodes, and dried overnight in vacuum conditions. A platinum wire and an Ag/AgCl (saturated KCl) electrode were used as counter electrode and reference electrode, respectively. The electrolyte solution was purged with high-purity nitrogen for 30 min and protected under nitrogen during the measurements. Methanol was electro-oxidized in an electrolyte containing 0.5 mol L^-1 ^H_2_SO_4 _and 2 mol L^-1 ^CH_3_OH in the potential range of -0.25 to 1.0 V at a sweep rate of 50 mV s^-1^.

## Results and discussion

### Characterization of Pt Ag alloy nanoisland/graphene hybrid composites

**Figure 1:**
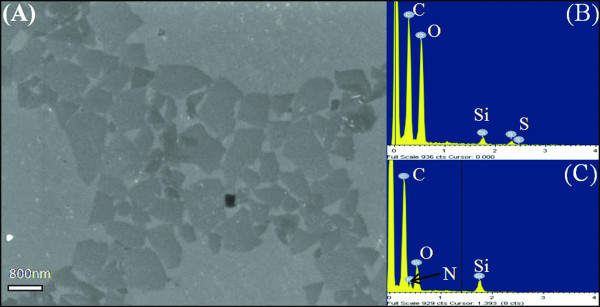
**(A), SEM image of graphene, (B), EDX analysis of GO, (C), EDX analysis of graphene**. Scale bar in **(A) **is 800 nm.

Figure 1 shows the SEM images of graphenes, EDX spectra of graphene oxide (GO), and graphene. In the course of graphene preparation, PVP was used and remarkably increased the stability of graphene sheets because of strong hydrophobic interactions between graphene sheets and PVP [10]. After reduction, the color of solution changed from yellow to dark black. Figure 1A shows that graphene sheets could self-assemble into a plane on silica wafer without coagulation. The width of graphene was about 800 nm. GO had an oxygen content of 43 atom%, as shown in Figure 1B, the atomic ratio of carbon to oxygen was 1.24. This result indicated there was more oxygen content than the empirical formula C_6_H_2_O_3 _proposed by Boehm [44]. After reduction, a nitrogen peak from PVP appeared in EDX spectra. Oxygen content in reduced graphene had two sources: one was from GO, the other one was from PVP. When evaluating GO's reduction degree, oxygen content came from PVP should be deducted. After first reduction, the atomic ratio of carbon to oxygen was 5.2, there was still 30% oxygen content remained (the EDX spectra was not shown). After second reduction, the atomic ratio of carbon to oxygen was 8.9, as shown in Figure 1C, only 14% oxygen content remained.

**Figure 2:**
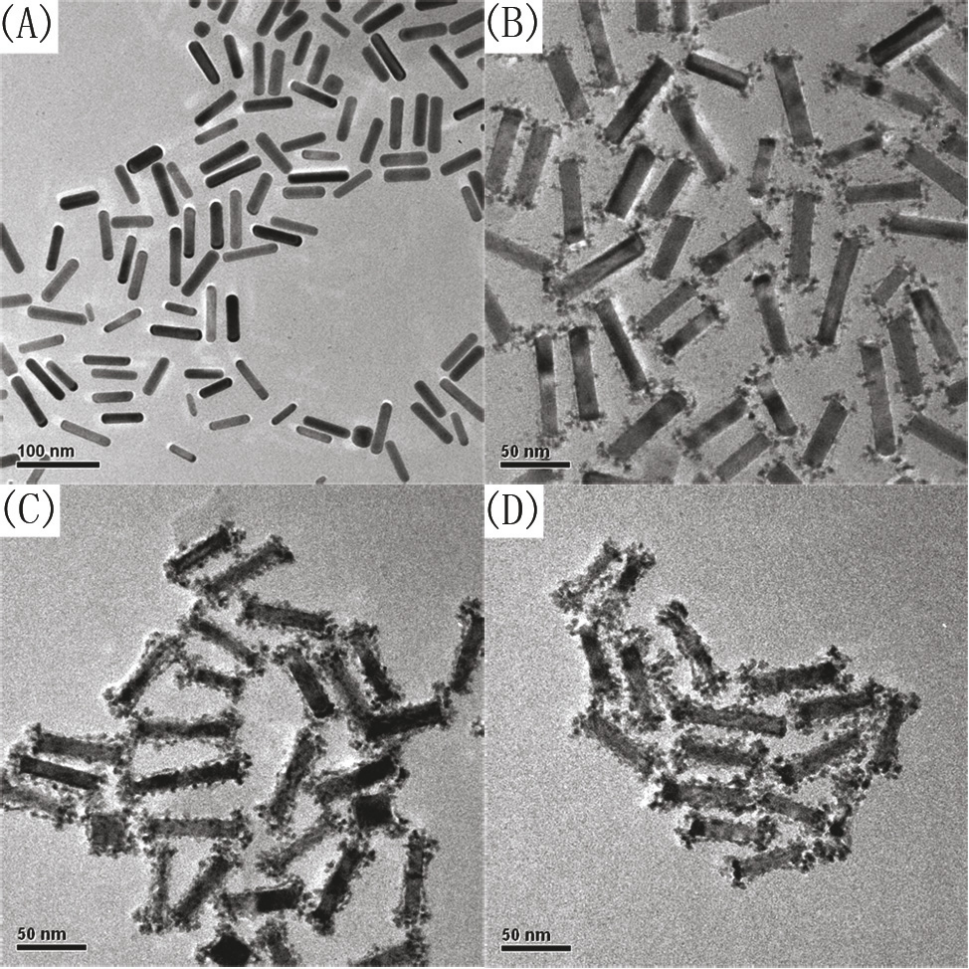
**TEM images of gold nanorods (Au NRs) (A), Au@Pt_0.34_Ag_0.66 _NRs (B), Au@Pt_0.57_Ag_0.43 _NRs (C), Au@Pt_0.64_Ag_0.36 _NRs (D)**. Scale bar in **(A) **is 100 nm, in **(B-D) **is 50 nm.

Figure 2 shows the TEM images of gold nanorods (Au NRs) and Au@AgPt alloy nanorods. Au NRs had a longitudinal surface plasmon resonance at 842 nm (see "Figure S1 in Additional file 1"). Both UV-Vis and TEM image indicate the prepared Au NRs had an aspect ratio of 4.4. Compared to Au NRs, all the three kinds of Ag-Pt alloy shell nanorods had rough surfaces. Ag-Pt alloy shell on the surface of Au NRs looked like nanodots or nanoislands. The nanoislands structure could increase surface area of Ag-Pt alloy shells, and improve the utilization of Pt material. When very few Pt^2+ ^ions were used, the nanodots of Ag-Pt alloy particles deposited almost on the two ends of Au NRs as shown in Figure 2B. With the amount of Pt^2+ ^ion increased, the nanodots of Ag-Pt alloy particles distributed uniformly on the surface of Au NRs. The amount of Ag and Pt in the shell layer was determined by EDX spectra. To mention the samples relatively easily, we used Au@Pt_*m*_Ag_*n *_to represent the samples. Here, *m *and *n *were percentage determined by EDX spectra.

**Figure 3:**
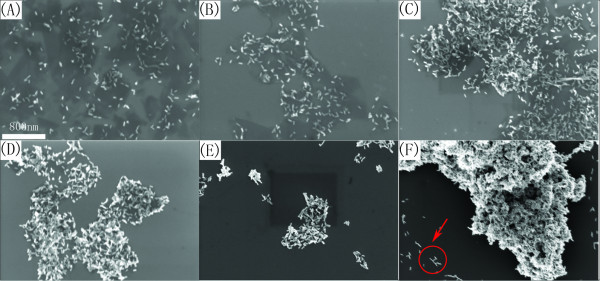
**SEM images of Au NRs/graphene hybrid composites with different weight ratios**: 1:1 **(A)**, 2:1 **(B)**, 5:1 **(C)**, 10:1 **(D)**, 20:1 **(E)**, 100:1 **(F)**. Scale bar: 800 nm.

Characterization of Au@PtAg alloy NRs/graphene hybrid composites was carried out by zeta potential test, SEM, and TEM. The zeta potential data were shown in Table 1. GO had a zeta potential of -64.2 mV, which is attributed to a large number of negatively charged functional groups such as carboxyl groups and hydroxyl groups. Prepared GO solution was good water soluble, and very stable at ambient condition because of electrostatic repulsion. After reduction, PVP-capped graphene sheets had a smaller negative zeta potential value. The zeta potential data of Au NRs and Au@PtAg NRs were, respectively, 30.4 and 44.8 mV, because of double-layer adsorption of CTAB. The larger value of Au@PtAg NRs was consistent with more surface area resulted from the islands structure. In a typical experiment of self-assembly, the aqueous dispersion of graphene sheets (0.5 mg mL^-1^) was mixed with Au NRs solution with different weight ratios (1:1, 1:2, 1:5, 1:10, 1:20, 1:100) and sonicated for 15 min to form a homogeneous mixture. Self-assembly of positively charged gold nanorods and Au@AgPt alloy nanorods with negatively charged graphene sheets resulted in formation of heavier entities; therefore, after 24 h, precipitation could be found at the bottom of the vessel. For the front four samples (the weight ratio of Au NRs to graphene 1:1, 1:2, 1:5, 1:10), the corresponding supernatants were colorless. By contrast, the corresponding supernatants of the last two samples were still red color which suggested extensive Au NRs used. As shown in Figure 3A, 3B (weight ratio 1:1 and 2:1), the edges of graphene sheets were quite clear, as well as Au NRs could spread out uniformly on silica wafer with few Au NRs found outside the graphene sheets; however, Au NRs adsorptive densities were very low. If a considerable quantity of Au NRs was used, in the case of weight ratio 20:1 and 100:1, redundant Au NRs could be found outside graphene sheets as marked by circles in Figure 3E, 3F. Moreover, the edges of graphene sheets could not be distinguished. When the weight ratio reached to 100:1, Au NRs deposited on graphene sheets by means of layer-by-layer, which lead to illegibility of the edges of graphene sheets. As the results shown in Figure 3C, 3D, the suitable weight ratio for self-assemble were 5:1 and 10:1, in which both graphene edges were clear, and Au NRs distributed uniformly on graphene sheets. Furthermore, the quantity of Au NRs loaded on graphene was appropriate.

**Table 1:** Average zeta potential measured at 25°C

	GO	GN	Au NRs	Au@Pt_0.57_Ag_0.43 _NRs
Zeta potential (mV)	-64.2	-39.6	30.4	44.8

TEM was also carried out for the sample of weight ratio 2:1 and 5:1 (see "Figure S2 in Additional file 1"). In the case of weight ratio 2:1, graphene could easily be recognized from the fringe and some pleats of graphene sheets (marked by red arrows). When weight ratio was 5:1, apart from uniformly distributed Au NRs, graphene sheets could not be seen clearly, which is because it was quite hard to make a distinction between them and the carbon-supported films on the copper grid due to the thin thickness of graphene sheets. SEM and TEM images both showed that self-assembly method was effective in producing homogeneous high-loading nanorods on the surface of graphene. The procedure of preparing graphene/Au@PtAg NRs hybrids was similar to that of graphene/Au NRs hybrids except for using Au@PtAg NRs as precursor for self-assembly. In the following experiment, we used the hybrid composition of weight ratio 5:1 for methanol electro-oxidation.

### Catalytic activity for methanol electro-oxidation

**Figure 4:**
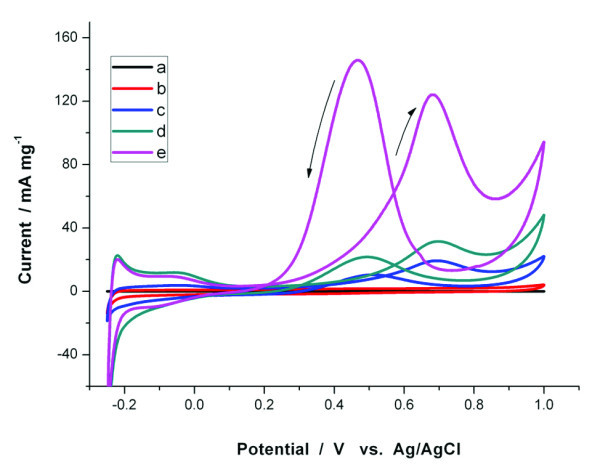
**Cyclic voltammetric curves for the electrooxidation of methanol (sweep rate: 50 mV s^-1^, 0.5 mol L^-1 ^H_2_SO_4_, 2 mol L^-1 ^CH_3_OH, 298 K) with the following electrocatalysts**. **(a) **graphene; **(b) **graphene/Au@Pt NRs; **(c) **graphene/Au@Pt_0.34_Ag_0.66 _NRs; **(d) **graphene/Au@Pt_0.57_Ag_0.43 _NRs; **(e) **graphene/Au@Pt_0.64_Ag_0.36 _NRs.

In recent years, DMFCs have intensely been studied because of their numerous advantages, which include high-energy density, the ease of handling a liquid, low operating temperature, and their possible applications to micro-fuel cells. Electrocatalytic materials restricted the performance and application of DMFCs. Herein, cyclic voltammetry (CV) was carried out to investigate the electrocatalytic activity of various graphene/Au@PtAg NRs hybrids materials for the oxidation of methanol. Three samples of Au@PtAg alloy nanorods and one sample of Au@Pt nanorods were used to prepare graphene hybrids materials and measured. In the blank control test, cyclic voltammetry was carried out in 0.5 mol L^-1 ^H_2_SO_4 _solution saturated with high-purity nitrogen gas to determine the hydrogen adsorption/desorption area between -0.3 and 0.1 V (see "Figure S3 in Additional file 1"). Hydrogen adsorption/desorption peak did not appear in CV curve of pure graphene. It revealed graphene could not adsorb hydrogen effectively in this case. As reported, Pt material is good catalyst in hydrogen adsorption/desorption and methanol electrooxidation. The results in "Figure S3 in Additional file 1" show that all the three samples of Au@PtAg alloy nanorods graphene hybrids materials and one sample of Au@Pt nanorods graphene hybrids materials had similar large hydrogen adsorption/desorption areas denoting similar effective electrochemical surface areas. Figure 4 shows cyclic voltammetric curves for the methanol electro-oxidation. For Au@Pt nanorods graphene hybrids materials (sample b), no obvious oxidation reduction peak was detected, indicating a poor catalytic performance for methanol electrooxidation. For the three samples of Au@PtAg alloy nanorods graphene hybrids materials (sample c, d, and e), methanol-oxidation peaks were clearly observed at about 0.69 V (versus Ag/AgCl) in the forward sweep and at 0.49 V in the backward sweep, respectively. The anodic peak current in the forward sweep was attributed to methanol electrooxidation, in the reverse sweep was attributed to the removal of the incompletely oxidized carbonaceous species formed in the forward sweep. These carbonaceous species were mostly in the form of linearly bonded Pt = C = O, which usually decreased catalytic activities of Pt materials and the so-called "catalyst poisoning." All PtAg alloy hybrids had good performance than pure Pt hybrids. The higher activity of PtAg alloy hybrids can be explained by the bifunctional mechanism [33-37,45] which was assumed that Ag promotes the oxidation of the strongly bound CO_ad _on Pt by supplying an oxygen source (Ag-OH_ad_). Among the five test samples shown in Figure 4, the sample graphene/Au@Pt_0.64_Ag_0.36 _NRs had the highest catalytic activity.

To gain more insights into the three catalysts, some electrochemical parameters such as electrochemically active surfaces (EAS) [40,45], utilization of Pt [40], catalytic activity [40], and the ratio of the forward oxidation current peak (*I*_f_) to the reverse current peak (*I*_b_), *I*_f_/*I*_b _[46-49] were calculated. EAS parameter provides important information regarding the number of available active sites. The EAS accounts not only for the catalyst surface available for charge transfer, but also includes the access of a conductive path to transfer the electrons to and from the electrode surface. Hydrogen adsorption/desorption in an electrochemical process is commonly used to evaluate the EAS. EAS could be obtained according to Equation 1, in which *Q*_H _is the charge consumed for the electrooxidation of adsorbed hydrogen; *Q*_e _is the elementary charge or charge of an electron; *A*_Pt _is the averaged atomic area of surface Pt atoms, which is 7.69 × 10^-2 ^nm^2 ^according to the atomic density of a Pt surface which is 1.3 × 10^19 ^m^-2^; and *W*_Pt _is the Pt loading at the working electrode. This equation is based on the well-established hydrogen-adsorption stoichiometry at a Pt surface (H: Pt = 1:1). Utilization of Pt was determined by Equation 2. *N*_t _is Pt atom loading on the working electrode; *N*_s _is utilizated Pt atom for electrooxidation [40]. *I*_f_/*I*_b _value could be used to evaluate the catalyst tolerance to the poisoning species. Low *I*_f_*/I*_b _value indicates poor oxidation of methanol to carbon dioxide during the anodic sweep and excessive accumulation of carbonaceous residues on the catalyst surface. High *I*_f_*/I*_b _value shows the converse case.

(1) EAS=(QHQe)APtWPt=APtQe×QHWPt

(2) $$U_{\text{pt}}=\frac{N_{\text{s}}}{N_{\text{t}}}=\frac{N_{\text{H}}}{N_{\text{t}}}$$

Electrochemical parameters (EAS, Pt utilization, catalytic activity, and *I*_f_*/I*_b_) of the three graphene/Au@PtAg NRs hybrids materials (sample c,d,e in Figure 4) were listed in Table 2. EAS and Pt utilization of the three graphene/Au@PtAg NRs hybrids catalysts were similar to that reported in previous reference listed in the fifth row. They showed much lower EAS and Pt utilization than that listed in the sixth row which reached nearly 100% Pt utilization. Interestingly, graphene/Au@Pt_0.64_Ag_0.36 _NRs (sample e) had high catalytic activity reached 124 mA mg Pt^-1^, which was just a bit lower than the sample of 99% Pt utilization in the sixth row. This result suggested graphene could enhance catalytic activity of Pt material. As Pt utilization was not high for our three samples tested in the experiment, if Pt utilization even enhanced, catalytic activity might even reach a new high platform. Furthermore, the ratio of *I*_f_*/I*_b _was all higher than the commercial E-TEK catalyst (0.74) [48]. It indicated that alloying with Ag can greatly improve the poisoning effect of Pt. As Ag content increased, anti-poisoning effect enhanced, but the catalytic activities decreased. The electrocatalytic stability of graphene/Au@Pt_0.64_Ag_0.36 _NRs (sample e) was tested by long-term repeated sweep by cyclic voltammetry in 0.5 mol L^-1 ^H_2_SO_4 _with 2 mol L^-1 ^CH_3_OH at 298 K (see "Figure S4 in Additional file 1"). We had done 200 sweep cycles for five times which lasted for about 15 h. The catalytic current behaved similar except for a little decrease in each 200 sweep cycles. For instance, in the first 200 sweep cycles, the catalytic current increased in the first 45 cycles. From the 45th to the 70th cycles, the catalytic current was stable at a high level, while it decreased afterward. In the period of decreased, the minimum value was still 60% of the maximum. In view of the four electrochemical parameters (EAS, Pt utilization, *I*_f_*/I*_b_, and sweep cycles), graphene/Au@Pt_0.64_Ag_0.36 _NRs (sample e) in this study is good electrode catalyst for methanol electro-oxidation.

**Table 2:** Utilization of Pt and the electrochemical properties of the Pt electrocatalysts

Catalyst	EAS (m^2 ^g^-1^)	*U*_Pt _(%)	Catalytic activity^a ^(mA mg Pt^-1^)	*I*_f_*/I*_b_
**1# **sample**c**	40.9	17.2	19.3	1.85
**2# **sample**d**	57.4	23.5	31.6	1.45
**3# **sample**e**	55.6	23.4	124	0.85
**Pt0.5^Au/C **[40]	28.1	12.0	11.6	
**Pt0.2^Au/C **[40]	58.1	24.7	26.2	
**Pt0.05^Au/C **[40]	233.3	99.2	162.5	

^a^For methanol oxidation, at 0.69 V.

**Figure 5:**
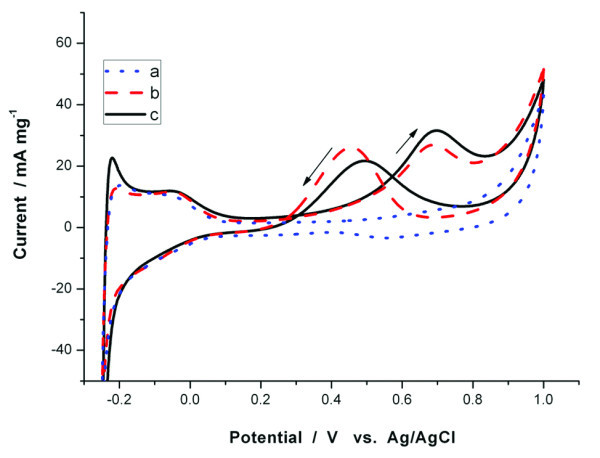
**Cyclic voltammetric curves for the electrooxidation of methanol (sweep rate: 50 mV s^-1^, 0.5 mol L^-1 ^H_2_SO_4_, 2 mol L^-1 ^CH_3_OH, 298 K)**. **(a) **the first cycle of Au@Pt_0.57_Ag_0.43 _NRs; **(b) **the 25th cycle of Au@Pt_0.57_Ag_0.43 _NRs; **(c) **the first cycle of graphene/Au@Pt_0.57_Ag_0.43 _NRs.

As mentioned above, graphene/Au@PtAg alloy NRs hybrid compositions were excellent materials for methanol electro-oxidation. To make out what role graphene played in the course, we done controlled experiment using pure Au@Pt_0.57_Ag_0.43 _NRs (sample a) and the NRs hybrid compositions of graphene and Au@Pt_0.57_Ag_0.43 _NRs (sample b), whose results were shown in Figure 5. In the case of the sample a (Au@Pt_0.57_Ag_0.43 _NRs without graphene), it was hard to find an oxidation peak in the first cycle (line a, blue dot line). With cycles went on, oxidation peak current gradually appeared and increased. The 25th cycle of sample a was shown in Figure 5 (line b, red dash line). As the results shown, it seemed that an electrical excitation process was needed to achieve a good oxidation current of methanol oxidation. In the reverse case, in the first cycle of sample b (Au@Pt_0.57_Ag_0.43 _NRs with graphene), obvious methanol-oxidation peaks were observed at 0.69 V in the forward sweep and at 0.49 V in the backward sweep (line c, black solid line), which were similar to that in the 25th cycle of sample a. For this reason, sample b had good oxidation current of methanol oxidation, and electrical excitation process was not needed.

Another important parameter to value catalytic activity of the samples is onset potential in electrical oxidation process. In forward sweep, all the samples had the same onset potentials (0.216 V). Otherwise, in backward sweep, sample b had frontier onset potentials (up to 124 mV) than sample a (without graphene). As mentioned above, the oxidation current of methanol oxidation in backward sweep represented the removal activity of the incompletely oxidized carbonaceous species (usually CO adsorbed on sample surface) generated in the forward sweep. The frontier onset potentials of graphene/Au@PtAg alloy NRs hybrid compositions indicated easier remove of the incompletely oxidized carbonaceous species. This phenomenon was very similar to that discovered by Yoo et al. before. In their research, Yoo et al. had done CO_ad _stripping voltammograms to explain the role graphene played in this reaction. The different state of CO adsorption on Pt/graphene was inferred to traditional Pt catalysts supported on carbon black [29]. In our study, the values of *I*_f_*/I*_b _were 1.46 and 1.24, respectively, for graphene/Au@PtAg alloy NRs hybrid compositions (the first sweep) and Au@PtAg alloy NRs (the 25th sweep) without graphene. The different onset potential and *I*_f_*/I*_b _value in backward sweep could be attributed to different CO adsorption state. The different CO adsorption state on graphene/Au@PtAg alloy NRs hybrid compositions and ordinary PtAg alloy NRs materials influenced the catalytic activity for methanol electrooxidation. Graphene in hybrid compositions could enhance anti-poisoning effect in the backward sweep. Graphene in the hybrid composition could change adsorption state of reactant, so the electrochemical process was affected. The higher oxidation peak in the first cycle of graphene/Au@PtAg alloy NRs hybrid compositions might result from the different interaction between graphene and methanol. Therefore, graphene in the hybrid compositions could improve the catalytic activity for methanol electrooxidation.

In addition, graphene had the advantages of good dispersion, high conductivity, large surface area, flexibility, and chemical stability. The higher catalytic activity of graphene architecture was attributed to the larger surface area which led to large currents and good dispersion of Au@PtAg NRs on the surface. The good dispersion of Au@PtAg NRs on graphene would give reactants easy access to the catalytic active sites, which would help to improve proton diffusion and mass transport.

## Conclusions

In this study, PtAg alloy nanoislands/graphene hybrid composites based on self-assembling of Au@PtAg NRs on graphene sheets were successfully prepared. The high-loading Au@PtAg NRs distributed uniformly on the surface of graphene sheets. It is confirmed that PtAg alloy nanoislands/graphene hybrid composites own better catalytic activity and longer stabilization for methanol oxidation compared with traditional method. Because large-scale graphene can be prepared by chemical reduction of graphene oxide; therefore, the PtAg alloy nanoislands/graphene hybrid composites can be obtained by large scale with low cost; therefore, as-prepared PtAg alloy nanoislands/graphene hybrid composite has great potential in applications such as electro-catalyst for DMFCs in near future.

## Competing interests

The authors declare that they have no competing interests.

## Authors' contributions

LF carried out the whole study. GG participated in the taking of SEM images. PH participated in the taking of TEM images. XW, CZ, JZ participated in the discussion of this research. DC and SG participated in the design of the study and gave instruction of the study. All authors read and approved the final manuscript.

## Supplementary Material

Additional file 1 **Figure S1. UV-Vis-NIR absorption spectra of the Au NRs**. **Figure S2. TEM images of Au NRs (A) and Au NRs/graphene hybrid composites with weight ratios**: 2:1 **(B)**, 5:1 **(C)**. Scale bar: 200 nm. **Figure S3. Cyclic voltammetric curves of the following electrocatalysts**: **(a) **graphene; **(b) **graphene/Au@Pt NRs; **(c) **graphene/Au@Pt_0.34_Ag_0.66 _NRs; **(d) **graphene/Au@Pt_0.57_Ag_0.43 _NRs; **(e) **graphene/Au@Pt_0.64_Ag_0.36 _NRs in 0.5 mol L^-1 ^H_2_SO_4 _solution at 298 K. **Figure S4. Stability of the graphene/Au@Pt_0.64_Ag_0.36 _NRs electrocatalyst over 200 cycles of methanol electrooxidation**. Click here for file
