# Supplementary material for: Preparation of Pt Ag alloy nanoisland/graphene hybrid composites and its high stability and catalytic activity in methanol electro-oxidation
Source: Nanoscale Res Lett. 2011 Oct 7;6(1):551. doi: 10.1186/1556-276X-6-551 (PMC3212089; doi:10.1186/1556-276X-6-551)
Supplement: Additional file 1 — Figure S1. UV-Vis-NIR absorption spectra of the Au NRs. Figure S2. TEM images of Au NRs (A) and Au NRs/graphene hybrid composites with weight ratios: 2:1 (B), 5:1 (C). Scale bar: 200 nm. Figure S3. Cyclic voltammetric curves of the following electrocatalysts: (a) graphene; (b) graphene/Au@Pt NRs; (c) graphene/Au@Pt0.34Ag0.66 NRs; (d) graphene/Au@Pt0.57Ag0.43 NRs; (e) graphene/Au@Pt0.64Ag0.36 NRs in 0.5 mol L-1 H2SO4 solution at 298 K. Figure S4. Stability of the graphene/Au@Pt0.64Ag0.36 NRs electrocatalyst over 200 cycles of methanol electrooxidation. [file 1556-276X-6-551-S1.DOC]

**Support information for**

Preparation of PtAg Alloy Nanoislands/Graphene Hybrid Composites and Its High Stable and Catalytic Activity of Methanol Electro-Oxidation

Lili Feng, Guo Gao, Peng Huang, Xiansong Wang, Chunlei Zhang, Jiali Zhang, Showu Guo, Daxiang Cui*

Key Laboratory for Thin Film and Microfabrication Technology of Ministry of Education, National Key Laboratory of Micro/Nano Fabrication Technology，Research Institute of Micro/Nano Science and Technology, Shanghai Jiao Tong University, Shanghai，200240, P. R. China

Correspondence should be addressed to: [dxcui@sjtu.edu.cn](mailto:dxcui@sjtu.edu.cn)

**Figure S1** UV-vis-NIR absorption spectra of the Au NRs.

**
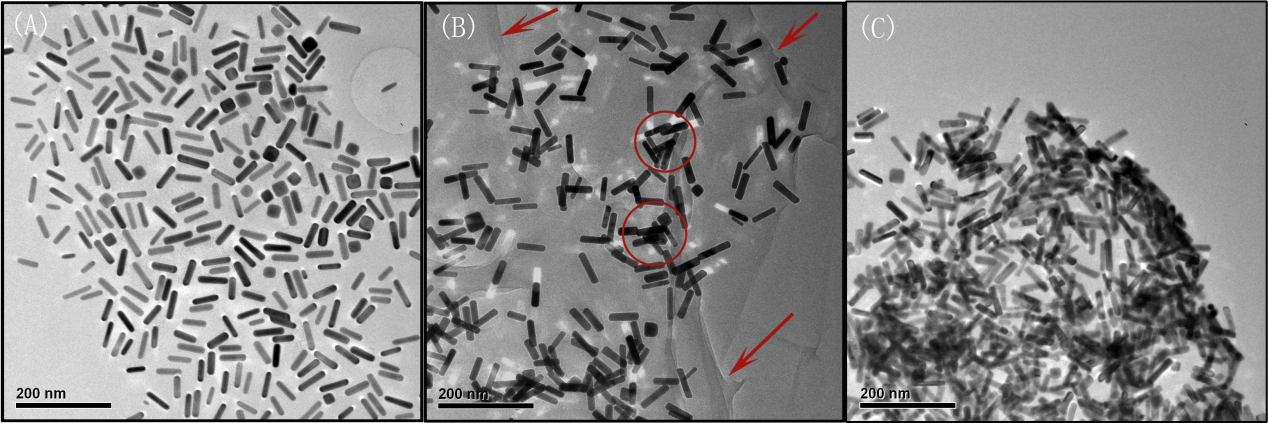
**

**Figure S2** TEM images of Au NRs(A) and Au NRs/graphene hybrid composites with weight ratios: 2:1(B), 5:1(C).Scale bar: 200 nm.

**Figure S3** Cyclic voltammetric curves of the following electrocatalysts:a) graphene; b)graphene/Au@Pt NRs; c) graphene/Au@Pt_0.34_Ag_0.66_ NRs; d) graphene/Au@Pt_0.57_Ag_0.43_ NRs; e) graphene/Au@Pt_0.64_Ag_0.36_ NRs in 0.5 mol L^-1^ H_2_SO_4_ solution at 298 K.

**Figure S4** Stability of the graphene/Au@Pt_0.64_Ag_0.36_ NRs electrocatalyst over 200 cycles of methanol electrooxidation.
